# Catalogue of the Library of the Surgeon General’s Office

**Published:** 1872-08

**Authors:** 


*Catalogue of the Library of the Surgeon General's Office, U. S. Army.* With an Alphabetical Index of Subjects.

*Library of the Surgeon General's Office, Supplement to Catalogue.* List of American Medical Journals.

At the commencement of the late war, the library of the Surgeon General's Office consisted of only about 350 volumes, since that time it has rapidly increased so that it now embraces over 13.000 volumes.

The establishment of a large Medical Library in a central locality where it can be open to the professional public, will meet a want which has long been felt by American Medical Men, who wishing to investigate some particular subject, have been put to considerable inconvenience in looking up the opinions of medical writers.

The printed catalogue before us will aid such very materially, showing them at a glance what books likely to treat of the subject under consideration are to be found in the library at Washington.

This library is still capable of improvement, and physicians can render much good to profession by fowarding copies of duplicate works which they may happen to possess, and are not to be found in the surgeon General's Office.

